# FGL2 is positively correlated with enhanced antitumor responses mediated by T cells in lung adenocarcinoma

**DOI:** 10.7717/peerj.8654

**Published:** 2020-03-13

**Authors:** Kai Yuan, Yanyan Feng, Hesong Wang, Lu Zhao, Wei Wang, Ting Wang, Yuyin Feng, Guangrui Huang, Anlong Xu

**Affiliations:** 1 School of Life Sciences, Beijing University of Chinese Medicine, Beijing, Beijing, China; 2 Division of Interdisciplinary Medicine and Biotechnology, Department of Medicine, Beth Israel Deaconess Medical Center/Harvard Medical School, Boston, MA, United States of America; 3 State Key Laboratory of Biocontrol, Department of Biochemistry, School of Life Sciences, Sun Yat-Sen (Zhongshan) University, Guangzhou, Guangdong, China

**Keywords:** *FGL2*, Lung cancer, Bioinformatics, Prognosis, Tumor infiltrating lymphocyte

## Abstract

Lung cancer is the most common malignant tumor, accounting for 25% of cancer-related deaths and 14% of new cancers worldwide. Lung adenocarcinoma is the most common type of pulmonary cancer. Although there have been some improvements in the traditional therapy of lung cancer, the outcome and prognosis of patients remain poor. Lung cancer is the leading cause of cancer-related deaths worldwide, with 1.8 million new cases being diagnosed each year. Precision medicine based on genetic alterations is considered a new strategy of lung cancer treatment that requires highly specific biomarkers for precision diagnosis and treatment. Fibrinogen-like protein 2 (FGL2) plays important roles in both innate and adaptive immunity. However, the diagnostic value of FGL2 in lung cancer is largely unknown. In this study, we systematically investigated the expression profile and potential functions of FGL2 in lung adenocarcinoma. We used the TCGA and Oncomine datasets to compare the *FGL2* expression levels between lung adenocarcinoma and adjacent normal tissues. We utilized the GEPIA, PrognoScan and Kaplan-Meier plotter databases to analyze the relationship between *FGL2* expression and the survival of lung adenocarcinoma patients. Then, we investigated the potential roles of *FGL2* in lung adenocarcinoma with the TIMER database and functional enrichment analyses. We found that *FGL2* expression was significantly lower in lung adenocarcinoma tissue compared with adjacent normal tissue. A high expression level of *FGL2* was correlated with better prognostic outcomes of lung adenocarcinoma patients, including overall survival and progression-free survival. *FGL2* was positively correlated with the infiltration of immune cells, including dendritic cells, CD8^+^ T cells, macrophages, B cells, and CD4^+^ T cells, in lung adenocarcinoma. Functional enrichment analyses also showed that a high expression level of *FGL2* was positively correlated with enhanced T cell activities, especially CD8^+^ T cell activation. Thus, we propose that high *FGL2* expression, which is positively associated with enhanced antitumor activities mediated by T cells, is a beneficial marker for lung adenocarcinoma treatment outcomes.

## Introduction

Lung cancer is a malignant lung tumor with unrestrained cell proliferation in the lung (Schwartz & Cote, 2016). As a highly prevalent and invasive disorder in women and men, more than 1.8 million people are diagnosed with lung cancer every year (Torre, Siegel & Jemal, 2016). Lung cancer accounts for almost 25% of cancer-related deaths and 14% of new cancers worldwide. Although there have been some improvements in the therapy and diagnosis of lung cancer, the outcome and prognosis of patients remain poor. The 5-year survival rate varies from 4–17% depending on regional and stage differences. Almost 1.6 million people die from lung cancer yearly. The lack of effective first-line drugs, the nonoptimal administration route and the formation of resistant tumors might be correlated with the poor prognostic survival of lung cancer patients (Pilkington et al., 2015).

Lung cancer is generally classified into two kinds of histopathology groups: non-small cell lung cancer and small cell lung cancer (Latimer, 2018). As the most common type of lung cancer, lung adenocarcinoma belongs to the non-small cell lung cancer group. Fifty percent of non-small cell lung cancer patients have a lung adenocarcinoma. It is also the most common form of lung cancer in Asian countries. The symptoms and signs of lung adenocarcinoma are similar to those of other types of lung cancer (Zappa & Mousa, 2016). Shortness of breath and persistent cough are the main symptoms of lung adenocarcinoma patients. The category of lung adenocarcinoma includes a variety of subtypes, such as acinar predominant adenocarcinoma, lepidic predominant adenocarcinoma, and micropapillary predominant adenocarcinoma. Radiotherapy, chemotherapy, surgical resection, and immunotherapy are common therapies employed to treat lung adenocarcinoma. However, because of the difficulties in diagnosing early lung adenocarcinoma, the average five-year survival rate is only approximately 18% (Hirsch et al., 2017).

Fibrinogen-like protein 2 (FGL2) plays important roles in both innate and adaptive immunity (Liu, Liu & Chen, 2017). The FGL2 protein is located on the surface of endothelial cells and macrophages (Yang & Hooper, 2013). CD8^+^ and CD4^+^ T cells constitutively secrete FGL2 protein to induce an inflammatory response. Several disorders, including severe acute respiratory syndrome (SARS), abortion and allograft rejection, are correlated with FGL2 (Hsieh et al., 2010; Zhao et al., 2013). In the area of cancer research, previous studies have found that altered *FGL2* gene expression contributes to immune surveillance evasion in murine renal carcinoma (Birkhäuser et al., 2013). Moreover, FGL2 contributes to glioblastoma multiforme (GBM) progression by stimulating immunosuppression mechanisms (Yan et al., 2015). However, the diagnostic value of FGL2 in lung cancer is largely unknown.

In this study, we systematically explored the potential roles of FGL2 in lung adenocarcinoma. Data downloaded from the TCGA dataset and PNAS were used to compare the *FGL2* expression levels between lung adenocarcinoma and adjacent normal tissues. Three bioinformatics databases, including GEPIA, PrognoScan and Kaplan–Meier plotter, were adopted to analyze the relationship of *FGL2* expression and the survival of lung adenocarcinoma patients. The TIMER database was used to discover the association between the immune status and *FGL2* expression in lung adenocarcinoma. Functional enrichment analyses, including Gene Ontology (GO), Kyoto Encyclopedia of Genes and Genomes (KEGG) pathway and GSEA, were used to explore the potential functions of FGL2 in lung adenocarcinoma development.

## Methods

### Bioinformatic evaluation of *FGL2* gene expression data

The normalized FPKM (fragments per kilobase per million mapped reads) values were downloaded from The Cancer Genome Atlas (TCGA) Data Portal (https://portal.gdc.cancer.gov). Normalized RNA-Seq datasets were used as input. Microarray mRNA data of lung adenocarcinoma were downloaded from Proc. Natl. Acad. Sci. USA (PNAS) (https://www.pnas.org/) (Bhattacharjee et al., 2001) and the GEO database (GSE32863). The microarray data were log2 transformed. *FGL2* expression was compared between lung cancer and normal adjacent tissues. Statistical significance was calculated with SPSS 20.0. Detailed information of included patients are listed in Table S1.

### Analysis of prognostic potential

The GEPIA, PrognoScan and Kaplan–Meier plotter databases were used to evaluate the prognostic potential of FGL2 in lung adenocarcinoma. The GEPIA (Gene Expression Profiling Interactive Analysis) database is a new web server (http://gepia.cancer-pku.cn/) for cancer and normal gene expression profiling and interactive analyses. GSEA was first introduced at 2003. Some concerns appeared immediately after GSEA was proposed (Tamayo et al., 2016). The concerns or limitations were list as follows: the null distribution of GSEA is superfluous and very hard to be worth calculating. The Kolmogorov–Smirnov-like statistic is not as sensitive as original. The results of GSEA are dependent on the algorithm clusters the genes, and the number of clusters being analyzed. The PrognoScan database is a new database (http://dna00.bio.kyutech.ac.jp/PrognoScan/) used to explore the relation between patient prognosis and gene expression with large collections of tumor microarray datasets. It is a useful platform to evaluate potential tumor markers in cancer research. The Kaplan–Meier plotter database (http://kmplot.com/analysis/) is a useful online tool used to assess the effects of specific genes on cancer prognosis and can estimate survival from lifetime data. Detailed information of included patients are listed in Table S1.

### TIMER database analysis

The TIMER database is a useful web tool that can be used to conduct a comprehensive analysis of tumor-infiltrating immune cells. This tool can evaluate the relationship between the immune status and *FGL2* mRNA expression in the lung adenocarcinoma microenvironment. The immune status includes inflammatory cells and the immune gene marker sets of immune cells. The TIMER database was used to measure the correlation between *FGL2* mRNA expression and the infiltration of immune cells, including B cells, CDT cells, CD4^+^ cells, macrophages and dendritic cells. Furthermore, the TIMER database was used to measure the correlation between *FGL2* mRNA expression and the immune gene marker sets of immune cells.

### Functional enrichment analysis

Gene Ontology (GO) and Kyoto Encyclopedia of Genes and Genomes (KEGG) analyses were used to analyze the potential function of FGL2 with the Database for Annotation, Visualization and Integrated Discovery (DAVID). GO analysis is a powerful bioinformatics tool used to determine biological processes (describing the physiological or cellular role carried out by the FGL2), cellular components (CC) and molecular functions (MF).

## Results

### The mRNA expression level of *FGL2* in lung adenocarcinoma

**Figure 1:**
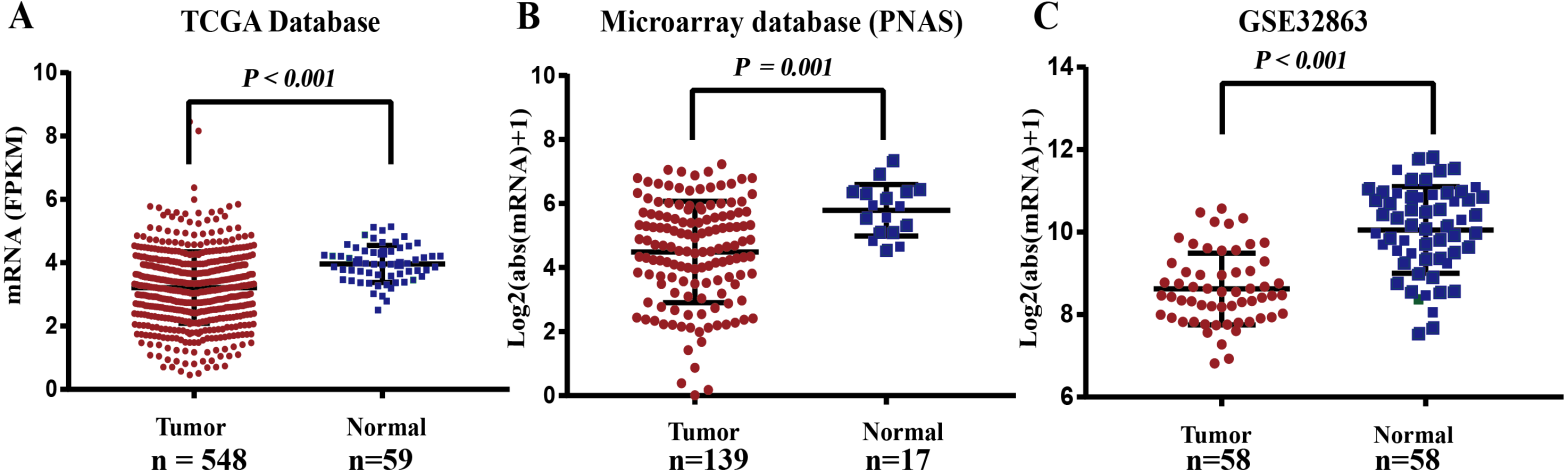
The mRNA expression level of *FGL2* in lung adenocarcinoma. (A) There was a significant difference between the FPKM value of *FGL2* mRNA expression in lung adenocarcinoma and normal adjacent tissue (*P* < 0.001). The left bar indicates the FPKM value of *FGL2* mRNA expression in lung adenocarcinoma tissue (mean FPKM value =3.958). The right bar indicates the FPKM value of *FGL2* expression level in normal adjacent tissue (mean FPKM value = 3.266). (B) The microarray database from PNAS showed a significant difference in the *FGL2* mRNA levels between lung adenocarcinoma and normal adjacent tissues (*P* = 0.001). (C) The GSE32863 database showed that the *FGL2* expression level in lung adenocarcinoma tissue was significantly lower than that in normal adjacent tissue (*P* < 0.001).

Related data were downloaded from the bioinformatics database TCGA Data Portal and used to compare the mRNA expression level of *FGL2* in lung adenocarcinoma and normal adjacent tissues. The results showed that the mean FPKM value of *FGL2* in lung adenocarcinoma was 3.266, which was significantly lower than that in normal adjacent tissue (3.958, *P* < 0.001) (Fig. 1A). Microarray data from PNAS and the GEO also showed that the *FGL2* mRNA expression level was lower in lung adenocarcinoma tissue than in normal adjacent tissue (Figs. 1B–1C).

### The prognostic value of *FGL2* in lung adenocarcinoma

**Figure 2:**
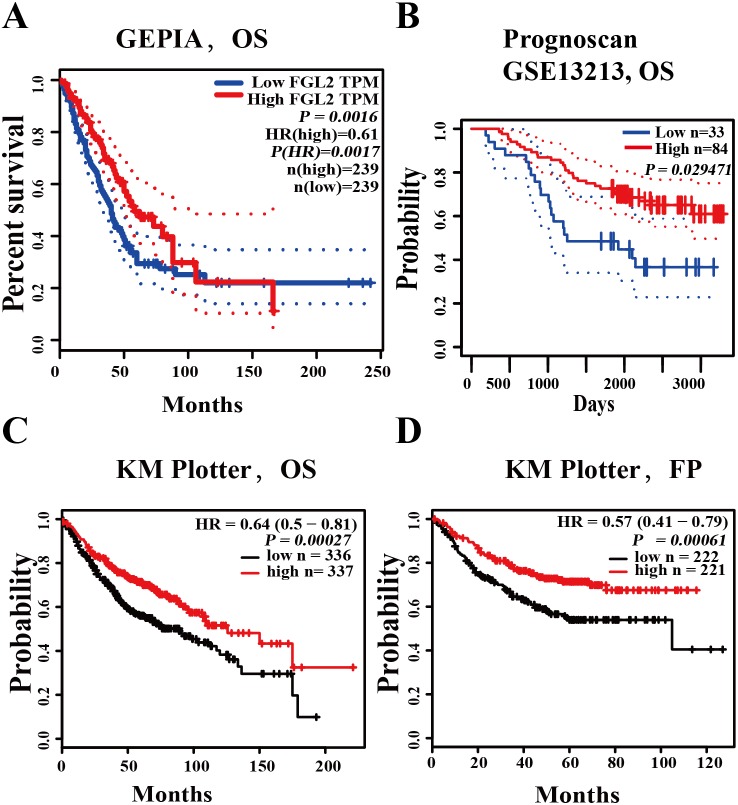
The prognostic value of *FGL2* in lung adenocarcinoma. (A) The GEPIA database showed that a high *FGL2* mRNA level was correlated with better overall survival (OS) in lung adenocarcinoma. (B) In the PrognoScan database, the cohort GSE13213 was used to evaluate the relationship between the *FGL2* expression level and overall survival in lung adenocarcinoma patients. (C–D) The Kaplan–Meier plotter database was used to analyze the relationship between the *FGL2* expression level and OS and progression-free survival in lung adenocarcinoma patients.

We used three databases (PrognoScan, GEPIA, Kaplan–Meier plotter) to analyze the prognostic value of *FGL2* in lung adenocarcinoma. In the GEPIA database, high *FGL2* expression was correlated with better overall survival (OS) in lung cancer (OS HR = 0.61, log-rank *P* = 0.0016, cutoff-high = 50%) (Fig. 2A). In the PrognoScan database, an analysis of the cohort GSE13213 showed that a high *FGL2* mRNA level was related to better overall survival in lung adenocarcinoma (OS HR = 0.68, 95% CI = 0.48 to 0.96, Cox *P* = 0.029471) (Fig. 2B). In the Kaplan–Meier plotter database, high *FGL2* mRNA expression was correlated with better overall survival and progression-free survival in lung adenocarcinoma patients (OS HR = 0.64, 95% CI = 0.50 to 0.81, log-rank *P* = 0.00027; FP HR = 0.57, 95% CI = 0.41 to 0.79, log-rank *P* = 0.00061) (Figs. 2C–2D). These results suggest that a high *FGL2* expression level is correlated better outcomes of lung adenocarcinoma.

### The correlation between immune cell infiltration and *FGL2* expression in lung adenocarcinoma

**Figure 3:**
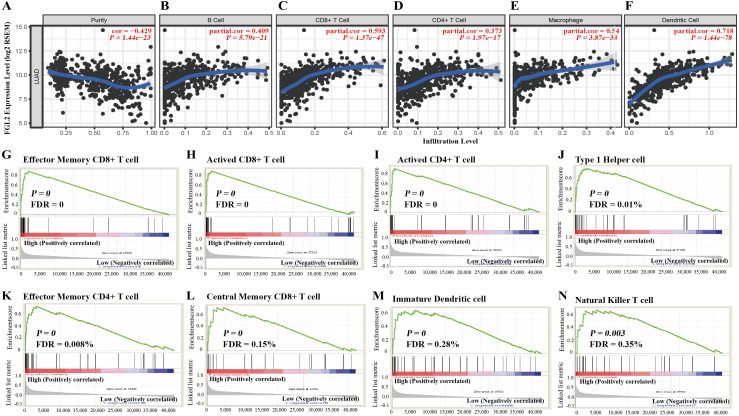
The correlation between immune cell infiltration and *FGL2* expression in lungadenocarcinoma. (A–F) The TIMER database showed that *FGL2* expression was positively correlated with the infiltration of B cells, CD8^+^ T cells, CD4^+^ T cells, macrophages, neutrophils and dendritic cells in lung adenocarcinoma. (G–N) GSEA showed that *FGL2* expression was positively correlated with effector memory CD8^+^ T cells, activated CD8^+^ T cells, activated CD4^+^ T cells, type 1 T helper cells, effector memory CD4^+^ T cells, central memory CD8^+^ T cells, immature dendritic cells and natural killer T cells.

We used the TIMER database to explore the correlation between immune cell infiltration and *FGL2* expression in lung adenocarcinoma. As shown in Fig. 3A, the *FGL2* expression level was positively correlated with B cell infiltration (*r* = 0.409, *P* = 5.79e−21), CD8^+^ T cell infiltration (*r* = 0.539, *P* = 1.37e−47), CD4^+^ T cell infiltration (*r* = 0.379, *P* = 1.97e−17), macrophage infiltration (*r* = 0.540, *P* = 3.87e−38) and dendritic cell infiltration (*r* = 0.718, *P* = 1.44e−78) in lung adenocarcinoma.

Then, we further investigated the correlation between the *FGL2* mRNA level and the subtypes of immune cell infiltration with GSEA (Fig. 3B). *FGL2* expression was positively correlated with the infiltration of effector memory CD8^+^T cells, activated CD8^+^ T cells, activated CD4^+^ T cells, type 1 T helper cells, effector memory CD4^+^T cells, central memory CD8^+^ T cells, immature dendritic cells and natural killer T cells.

### The correlation between the immune marker sets of immune cells and *FGL2* expression in lung adenocarcinoma

To validate the relationship between *FGL2* expression and immune cell infiltration, we also investigated the correlation between the immune marker sets of immune cells and *FGL2* expression in lung adenocarcinoma with the TIMER and GEPIA databases. As shown in Table 1, data from the TIMER database indicated that *FGL2* expression was positively correlated with most of the immune marker sets. For example, *FGL2* was positively corelated with the T cell gene markers *CD3D* (Cor = 0.532, *P* = 1.97e−37), *CD3E* (Cor = 0.583, *P* = 3.25e−46), and *CD2* (Cor = 0.646, *P* = 1.59e−59); CD8^+^ T cell (cytotoxic T lymphocyte, CTL) gene markers *CD8A* (Cor = 0.561, *P* = 3.93E−42) and *CD8B* (Cor = 0.456, *P* = 1.03E−26), dendritic cell gene markers *HLA-DPB1* (Cor = 0.635, *P* = 6.07E−57), *HLA-DRA* (Cor = 0.683, *P* = 6.79E−69), and *HLA-DPA1* (Cor = 0.681, *P* = 1.45E−68); and Th17 gene markers *ICOS* (Cor = 0.667, *P* = 1.00E−64) and *IL1B* (Cor = 0.428, *P* = 2.26E−23).

**Table 1:** Correlation between immune marker sets of immune cells and *FGL2* expression in lung adenocarcinoma based data from TIMER database.

Description	Gene markers	Cor	*P*	Description	Gene markers	Cor	*P*
T cell (general)	CD3D	0.532	1.97E−37	CTL (Cytotoxic T Lymphocytes )	CD8A	0.561	3.93E−42
	CD3E	0.583	3.25E−46		CD8B	0.456	1.03E−26
	CD2	0.646	1.59E−59		GZMB	0.325	1.45E−13
B cell	CD19	0.232	2.01E−07	Dendritic cell	HLA-DPB1	0.635	6.07E−57
	CD79A	0.21	2.54E−06		HLA-DQB1	0.386	5.34E−19
	CD79B	0.316	7.03E−13		HLA-DRA	0.683	6.79E−69
	CD22	0.314	7.18E−15		HLA-DPA1	0.681	1.45E−68
M1 Macrophage	INOS	0.039	3.85E−01		DEC-205	0.471	1.19E−28
	CIITA	0.544	2.73E−39		BDCA-1	0.424	6.01E−23
	IRF5	0.307	3.39E−12		BDCA-4	0.257	6.66E−09
	COX2	−0.127	4.72E−03		BDCA-2	0.48	8.93E−30
M2 Macrophage	CD163	0.618	2.61E−53		CD11c	0.46	3.07E−27
	IRF4	0.375	6.49E−18	Th1	CD38	0.211	2.18E−06
	VSIG4	0.604	3.00E−50		T-bet	0.48	8.15E−30
	MS4A4A	0.685	1.40E−69		STAT4	0.409	2.78E−21
TAM	CCL2	0.35	1.16E−15		STAT1	0.461	2.63E−27
	CCL5	0.506	2.07E−33		IFN-γ	0.432	7.89E−24
	CD68	0.554	4.95E−41		TNF-α	0.295	2.51E−11
	IL10	0.603	3.42E−50	Th2	GATA3	0.313	1.22E−12
Neutrophils	CD66b (CEACAM8)	0.237	1.06E−07		IL13	0.142	1.63E−03
	CD15	0.135	2.74E−03		STAT6	0.077	8.69E−02
	CD11b (ITGAM)	0.587	5.45E−47	Tfh	BCL6	−0.03	5.09E−01
	CCR7	0.434	4.99E−24		CD200	0.275	5.64E−10
Natural killer cell	NKp46	0.405	7.42E−21		IL21	0.319	4.27E−13
	NKp44	0.109	1.52E−02		ICOS	0.667	1.00E−64
	NKp30	0.481	7.29E−30	Th17	STAT3	0.035	4.39E−01
	FCGR3A	0.682	1.36E−68		IL17A	0.183	4.19E−05
	FCGR3B	0.326	1.13E−13		IL1A	0.227	3.36E−07
	NKG2A (KLRC1)	0.351	1.01E−15		IL1B	0.428	2.26E−23
	KIR2DL1	0.151	7.73E−04		CCL20	−0.052	2.53E−01
	KIR2DL3	0.22	7.87E−07	Treg	FOXP3	0.456	1.04E−26
	KIR3DL1	0.169	1.64E−04		CCR8	0.572	3.47E−44
					TGFβ	0.365	5.36E−17

Data from the GEPIA database also showed similar results to that of the TIMER database. Detailed information is listed in Table 2.

**Table 2:** Correlation between immune marker sets of immune cells and *FGL2* expression in lung adenocarcinoma based data from GEPIA database.

Description	Gene markers	Cor	*P*	Description	Gene markers	Cor	*P*
T cell (general)	CD3D	0.500	0	CTL (Cytotoxic T Lymphocytes )	CD8A	0.530	0
	CD3E	0.580	0		CD8B	0.390	0
	CD2	0.640	0		GZMB	0.280	3.50E−10
B cell	CD19	0.240	1.30E−07	Dendritic cell	HLA-DPB1	0.510	0
	CD79A	0.220	1.70E−06		HLA-DQB1	0.230	3.00E−07
	CD79B	0.290	1.70E−10		HLA-DRA	0.610	0
	CD22	0.290	1.50E−10		HLA-DPA1	0.610	0
M1 Macrophage	INOS	0.040	3.80E−01		DEC-205	0.450	0
	CIITA	0.570	0		BDCA-1	0.290	1.10E−10
	IRF5	0.300	3.70E−11		BDCA-4	0.250	2.90E−08
	COX2	−0.110	2.00E−02		BDCA-2	0.420	0
M2 Macrophage	CD163	0.460	0		CD11c	0.410	0
	IRF4	0.300	1.70E−11	Th1	CD38	0.160	3.30E−04
	VSIG4	0.500	0		T-bet	0.130	3.00E−03
	MS4A4A	0.610	0		STAT4	0.320	3.30E−13
TAM	CCL2	0.300	2.40E−11		STAT1	0.510	0
	CCL5	0.430	0		IFN-γ	0.470	0
	CD68	0.550	0		TNF-α	0.320	6.90E−13
	IL10	0.580	0	Th2	GATA3	0.017	7.10E−01
Neutrophils	CD66b (CEACAM8)	0.057	2.10E−01		IL13	0.240	8.30E−08
	CD15	0.110	1.50E−02		STAT6	0.100	2.50E−02
	CD11b (ITGAM)	0.560	0	Tfh	BCL6	0.085	6.10E−02
	CCR7	0.410	0		CD200	0.370	0
Natural killer cell	NKp46	0.390	0		IL21	0.560	0
	NKp44	0.068	1.40E−01		ICOS	0.650	0
	NKp30	0.440	0	Th17	STAT3	0.210	2.90E−06
	FCGR3A	0.660	0		IL17A	0.220	8.70E−07
	FCGR3B	0.430	0		IL1A	−0.003	9.50E−01
	NKG2A (KLRC1)	0.230	5.00E−07		IL1B	0.380	0
	KIR2DL1	−0.054	2.30E−01		CCL20	−0.046	3.10E−01
	KIR2DL3	0.130	5.70E−03	Treg	FOXP3	0.500	0
	KIR3DL1	0.150	1.30E−03		CCR8	0.580	0
					TGFβ	0.330	1.80E−13

### Functional enrichment analyses of *FGL2*-correlated genes

**Figure 4:**
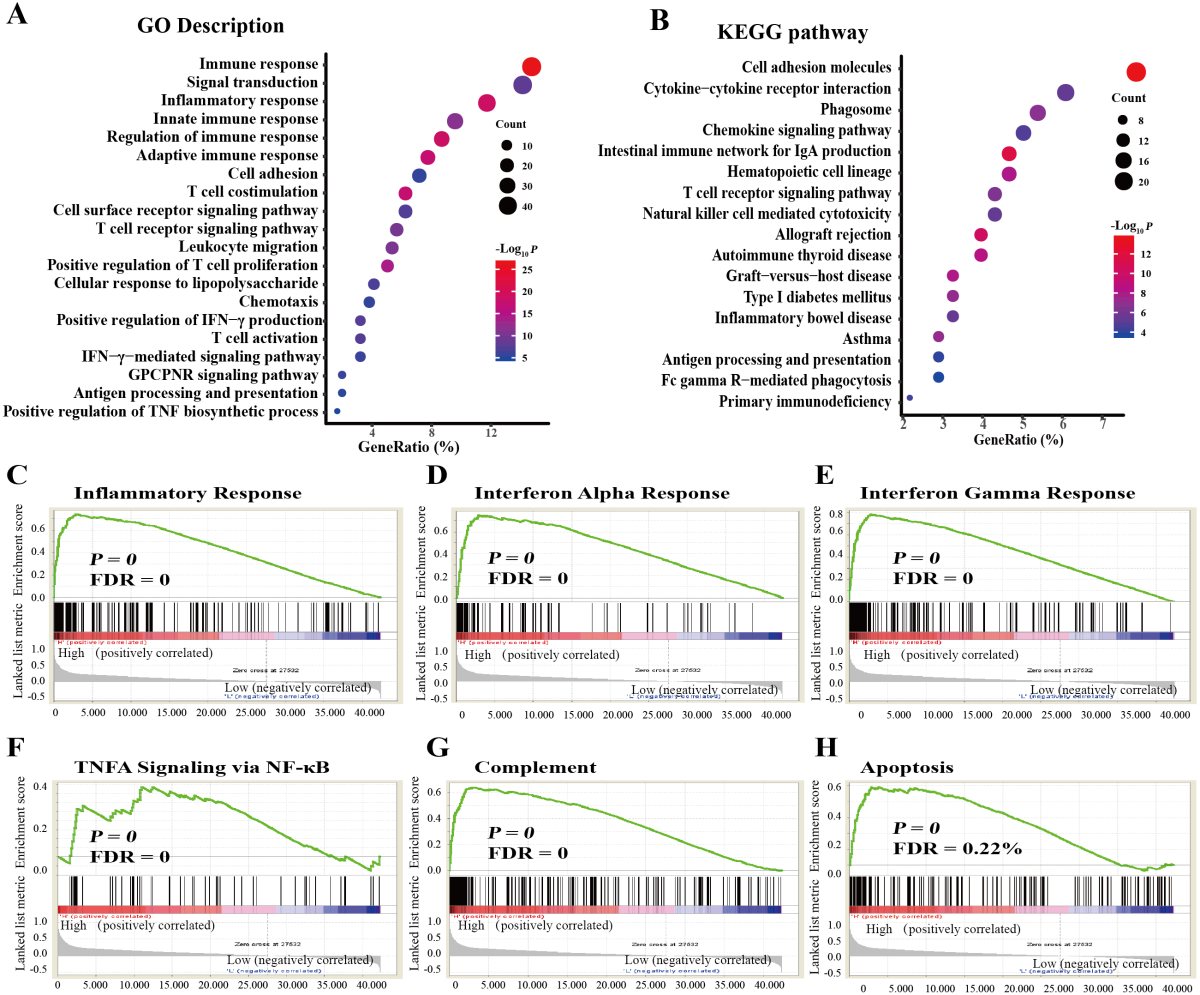
Functional enrichment analysis of *FGL2*-correlated genes in lung adenocarcinoma. (A) GO analysis of *FGL2*-correlated genes in lung adenocarcinoma with the TCGA dataset. (B) KEGG analysis of *FGL2*-correlated genes in lung adenocarcinoma with the TCGA dataset. (C–H) GSEA was performed to explore the biological functions of *FGL2* in lung adenocarcinoma.

We performed GO and KEGG pathway analyses with data obtained from the TCGA dataset. GO analysis indicated that *FGL2*-correlated genes were enriched in the immune response, the adaptive immune response, the positive regulation of T cell proliferation, the positive regulation of interferon-gamma production, the positive regulation of tumor necrosis factor production, T cell activation, the interferon-gamma-mediated signaling pathway, T cell costimulation, T cell differentiation, the T cell receptor signaling pathway, antigen processing and presentation of exogenous peptide antigen via MHC class I, TAP-dependent, etc (Fig. 4A). KEGG pathway analysis showed that *FGL2* was correlated with genes involved in cell adhesion, natural killer cell-mediated cytotoxicity, the TNF signaling pathway, the T cell receptor signaling pathway, antigen processing and presentation, etc. (Fig. 4B).

We also conducted GSEA to explore the biological functions of FGL2 in lung adenocarcinoma. *FGL2* expression was positively related to the inflammatory response, the interferon alpha response, the interferon gamma response, TNF alpha signaling via NF-κB, complement, and apoptosis (Fig. 4C).

## Discussion

Lung adenocarcinoma is a type of non-small cell lung cancer and the most common histologic type of lung cancer. In lung adenocarcinoma, there is a variety of divergent molecular, pathologic, and clinical spectra (Testa, Castelli & Pelosi, 2018). The common signs of lung adenocarcinoma include weight loss, dyspnea, chest pain, and cough. The extrapulmonary manifestations include hypercalcemia of the malignancy and hypertrophic pulmonary osteoarthropathy. Tobacco smoking is a risk factor for lung adenocarcinoma (Song et al., 2017). In addition to smoking, gene mutations are also important mutagenic factors of lung adenocarcinoma (Ding et al., 2008). Somatic mutations might influence tumor suppressor genes and oncogenes in lung adenocarcinoma. Recent opinions on the treatment of lung adenocarcinoma have changed from traditional chemotherapy to precision medicine based on the genetic alterations of cancer (Herbst, Morgensztern & Boshoff, 2018). Previous studies have found that many gene mutations contribute to lung adenocarcinoma. The mutated genes include *EGFR*, *KRAS*, *TP53*, *STK11*, *NF1*, and *KEAP1*. The EGFR-activating mutation frequency varies depending on ethnicity and region. EGFR is correlated with the cell proliferation, invasion, survival, and angiogenesis of tumors (Sharma et al., 2007). Oral EGFR tyrosine kinase inhibitors such as gefitinib and erlotinib prolonged progression-free survival and the objective response rate compared with traditional chemotherapy (Mok et al., 2009; Shepherd et al., 2005). The inactivation of KEAP1 in KRAS mutations is related to the inhibition of glutaminase in lung cancer (Romero et al., 2017). TP53 mutations are commonly found in advanced-grade lung adenocarcinoma  (Ahrendt et al., 2003). Precision medicine also provides genes that are beneficial for the progression and survival of cancers. FGL2 is an important factor in regulating the immune system. FGL2 is upregulated in GBM, promoting GBM development by suppressing dendritic cell activities (Yan et al., 2015). However, the diagnostic value of FGL2 in lung cancer is largely unknown. In this study, we systematically investigated the expression profile and potential functions of FGL2 in lung adenocarcinoma.

First, we evaluated the expression of *FGL2* in lung adenocarcinoma tissue and adjacent normal tissue. Data obtained from the TCGA dataset and the Oncomine database indicated that *FGL2* expression was significantly lower in lung adenocarcinoma tissue than in adjacent normal tissue. This implied that FGL2 might be a beneficial biomarker of lung adenocarcinoma.

Second, we analyzed the relationship of the *FGL2* level and the prognostic survival of lung adenocarcinoma patients with three bioinformatics datasets. A high *FGL2* mRNA level was correlated with better prognostic outcomes of lung adenocarcinoma, including overall survival and progression-free survival. These results indicate that FGL2 might negatively regulate the progression of lung adenocarcinoma.

Then, we explored the correlation between *FGL2* expression and the immune status in the tumor microenvironment of lung adenocarcinoma. *FGL2* expression was positively correlated with immune cell infiltration and immune marker sets in lung adenocarcinoma. The immune cells included CD8^+^ T cells, CD4^+^ T cells, macrophages, B cells and dendritic cells. CD8^+^ T cells (often called cytotoxic T lymphocytes or CTLs) are very important for tumor surveillance. CD8^+^ T cells use three major mechanisms to kill tumor cells. The first is the secretion of cytokines (primarily TNF-α and IFN-γ). Our results showed that high *FGL2* expression was positively correlated with IFN-γ production and signaling and TNF-α production and signaling. The second major function is the production and release of cytotoxic granules, which mainly contain perforin 1 (PRF1) and granzymes. Our results showed that the expression level of *FGL2* was positively correlated with the expression levels of *PRF1*, *granzyme K* (*GZMK*), *GZMA*, *GZMH*, *GZMB*, and *GZMM*. The third antitumor function of CD8^+^ T cells is to induce the apoptosis of tumor cells via Fas/Fas ligand (FasLG) interactions. Our results showed that the expression level of *FGL2* was positively correlated with the expression levels of *Fas* and *FasLG*. These results show that high *FGL2* expression is closely related to enhanced CD8^+^ T cell-mediated antitumor activities. Dendritic cells (DCs) are considered important factors that provide protective immunity against lung adenocarcinoma (Wang, Huang & Li, 2019). Inactive DCs are correlated with the poor prognosis of lung cancer patients. DCs present antigens to activate antitumor T cells. Mature DCs in lung cancer express high levels of cytokines and costimulatory molecules (CD40/80/86) to activate T cells (Macri et al., 2018). MHC type II molecules on DCs promote the activation of CD4^+^ T cells. MHC type 1 molecules on DCs promote the activation of CD8^+^ T cells (Liu & Cao, 2015). Except for activating T cells, DCs can also recruit and activate NK cells by secreting C-C chemokine receptor type 5 (CCR5) at the tumor site (Liu et al., 2008). In our study, we found that *FGL2* expression was positively corelated to the infiltration of CD4^+^ T cells, CD8^+^ T cells, macrophages, B cells and DCs in lung adenocarcinoma. In addition, the *FGL2* level was positively correlated with several subtypes of immune cells, including effector memory CD8^+^ T cells, activated CD8^+^ T cells, activated CD4^+^ T cells, type 1 T helper cells, effector memory CD4^+^ T cells, central memory CD8^+^ T cells, immature dendritic cells and natural killer T cells. These results indicate that FGL2 plays an important role in antitumor immunity by enhancing antitumor activities in lung adenocarcinoma.

There were some low correlation values for certain gene markers assayed to ascertain correlation between immune markers and *FGL2* expression. As for B cells, Table 1 indicated the correlation of B cells gene markers varied from 0.21–0.316. In Table 2, the correlation of B cells gene markers varied from 0.24–0.29. In addition, Fig. 3A indicated the correlation between *FGL2* and B cells was 0.409. The results showed that *FGL2* has not possessed strong correlation with B cells in the tumor microenvironment of lung adenocarcinoma. Tumor infiltrating B cells appear in every stage of lung cancer and play critical roles in shaping tumor progression. However, the B cells functions in antitumor immunity of lung cancer are controversial (Wang et al., 2019). Some studies demonstrated that tumor infiltrating B cells have protective effects on anti-tumor immunity in lung cancer, while other studies revealed that tumor infiltrating B cells have inhibitory effects on antitumor immunity in lung cancer. Owning to the B cells functions in antitumor immunity is dualistic, *FGL2* might possessed poor or moderate correlation with B cells in the tumor microenvironment of lung adenocarcinoma. Apart from B cells, Fig. 3A showed correlation between *FGL2* and CD4^+^ T cells was 0.373. This result indicated that the correlation between *FGL2* and CD4^+^ T cells was moderate at best. Previous studies found that the role of CD4^+^ T cells in antitumor activity of lung cancer is dualistic (Zheng, Hu & Yao, 2017). Some types of CD4^+^ T cells impair the functions of cytotoxic T lymphocytes to promote the tumor development, while some types of CD4^+^ T cells induce the activation of cytotoxic T lymphocytes to exert antitumor immune response. This might explain the reason that correlation between *FGL2* and CD4^+^ T cells was poor and moderate in lung adenocarcinoma.

Finally, we performed functional enrichment analysis to explore the biological functions of FGL2 in lung adenocarcinoma. GO and KEGG analyses indicated that *FGL2*-correlated genes are mainly enriched in pathways involved in T cell proliferation/differentiation/activation and antigen processing and presentation. GSEA showed that *FGL2* expression was positively correlated with enhanced tumor killing. These results further indicate that FGL2 enhances antitumor activities in lung adenocarcinoma.

In the research conducted by Zhu et al. titled “Stroma-derived fibrinogen-like protein 2 activates cancer-associated fibroblasts to promote tumor growth in lung cancer”, they found that FGL2 could promote tumor growth of lung cancer by activating cancer-associated fibroblasts using a mouse model of Lewis lung carcinoma (Zhu et al., 2017). In the research conducted by Yan et al. (2019) titled “FGL2 promotes tumor progression in the CNS by suppressing CD103^+^ dendritic cell differentiation”, they found FGL2 accelerated tumor progression of GBM by suppressing CD103^+^ dendritic cell differentiation. In our study, we extracted data of lung adenocarcinoma patients from different clinical databases such as TCGA, PrognoScan, and TIMER. We found that *FGL2* expression was significantly lower in lung adenocarcinoma tissue compared with adjacent normal tissue. A high expression level of *FGL2* was correlated with better prognostic outcomes of lung adenocarcinoma patients. We speculated that FGL2 might play differential roles in distinct models of cancer.

In the mouse model of Lewis lung carcinoma, FGL2 induced an activated and pro-tumorigenic phenotype of cancer-associated fibroblasts in the tumor microenvironment (TME). Cancer-associated fibroblasts, also known as tumor-associated fibroblast, promote tumorigenic features by producing cytokines, or initiating extracellular matrix remodeling in the tumor microenvironment (Erdogan & Webb, 2017). The cytokines could disrupt normal cell functions, such as normal cell cycle regulation to active their pro-tumor actions (Öhlund, Elyada & Tuveson, 2014). In addition, cancer-associated fibroblasts produce and secret angiogenic factors such as fibroblast growth factor (FGF), and vascular endothelial growth factor (VEGF) to stimulate angiogenesis supporting the formation of tumors and the proliferation of cancer cells and metastasis (Shiga et al., 2015).

In the mouse model of brain tumor, Yan et al. demonstrated that FGL2 promotes GBM tumor progression by suppressing CD103^+^ dendritic cell differentiation. The function of dendritic cells and their subtypes in GBM has not been elucidated clearly (Srivastava et al., 2019). Dendritic cells might interplay with other types of immune cells including macrophages, T cells, and microglia in the tumor microenvironment (TME). Some researchers considered that certain subsets of dendritic cells recognize and present tumor antigens to induce the T cells immune responses (D’Agostino et al., 2012). If the CD103^+^ dendritic cell differentiation was suppressed, the antigen processing functions of dendritic cells might be impaired in GBM. So, FGL2 might promote GBM tumor progression by suppressing CD103^+^ dendritic cell differentiation.

In our study, we demonstrated *FGL2* was positively correlated with T cells, especially CD8^+^T cells activation in the tumor microenvironment of lung adenocarcinoma patients. CD8^+^T cells, also known as cytotoxic T lymphocytes (CTLs), play important role of antitumor immune response in the tumor microenvironment (Aerts & Hegmans, 2013). CTLs could recognize and kill tumor cells by the complex formation of T-cell receptor (TCR) and human leukocyte antigen class (HLA). TCR and related signaling molecules could activate the transduction cascade to induce immune synapse and stimulate antitumor responses of CTLs (Farhood, Najafi & Mortezaee, 2019). Then, the activated CTLs produce and secret cytotoxic granules such as granzymes and perforin into the targeted tumor cells. Adhesive and co-stimulatory molecules including CD80/CD86, CD11a/CD18, and lymphocyte function-associated antigen 1 (LFA-1) are important in the process of TCR-mediated CTLs antitumor effects (Durgeau et al., 2018). CTL-associated antigen 4 (CTLA-4) and programmed death-1 receptor (PD-1)-ligand (PD-L1) are checkpoint receptors could be targeted to relieve CTLs exhaustion. Antigen presenting cells (APCs) such as dendritic cells (DCs) and macrophages could activate naïve CD8^+^ T cells by binding the TCRs with CD3 and other costimulatory molecules. FGL2 is mainly expressed in APCs such as DCs and macrophages. So, we infer that FGL2 might activate CTLs functions to exert anti-tumor effects by stimulating APCs to bind naïve CD8^+^ T cells.

In brief, FGL2 might play different roles in different types of cancer models. In lung cancer animal model conducted by Zhu et al., FGL2 might promote tumor progress by activating cancer-associated fibroblasts in tumor microenvironment. In GBM animal model, FGL2 promotes GBM progress by suppressing CD103^+^ dendritic cell differentiation. In clinical databases of lung cancer patients, FGL2 exhibited antitumor activities by activating CTLs in the tumor microenvironment of lung cancer.

There are no significant differences in the expression levels of *FGL2* between patients with or without EGFR or KRAS mutations in the TCGA database. There is no significant correlation between *FGL2* and other genes implicated in lung adenocarcinoma such as *EGFR*, *KRAS*, *TP53*, *STK11*, *NF1*, and *KEAP1*. We speculated that FGL2 might indirectly affect those genes in lung adenocarcinoma by changing the immune status in the tumor environment. EGFR regulates several signaling transduction cascades such as MAPK, JNK, and Akt signaling pathways, leading to tumor cell proliferation, cell cycle progression, angiogenesis, and metastasis (Bethune et al., 2019). EGFR-targeted therapy such as EGFR tyrosine kinase inhibitors (EGFR-TKIs) alters the tumor microenvironment in lung cancer (Matsumoto et al., 2019). EGFR-TKIs could increase cytotoxic CD8^+^ T cells and dendritic cells in the tumor environment of lung cancer (Jia et al., 2019). FGL2 also increase cytotoxic CD8^+^ T cells and dendritic cells in the tumor environment of lung cancer. So, FGL2 might affect EGFR by influencing immune status in tumor environment of lung cancer. KRAS is important in promoting cell survival and growth in tumor cells. Almost 30% patients with lung adenocarcinoma are positive for KRAS gene mutation (Tomasini et al., 2016). KRAS is a strong initiator of tumorigenesis in lung adenocarcinoma. It is also a predictive response to targeted therapy of lung adenocarcinoma (Dias Carvalho et al., 2018). KRAS is related with immune-suppressed state by regulating components of adaptive and innate immune response (Cullis, Das & Bar-Sagi, 2018). Mutant KRAS could up-regulate immunosuppressive cells in tumor such as myeloid-derived suppressor cells (MDSCs), CD4^+^FoxP3^+^ T regulatory cells, and CD19^+^IL10^+^ B regulatory cells. These immunosuppressive cells could suppress the activities of tumoricidal cells such as CD8^+^ T cytotoxic cells, natural killer (NK) cells in the tumor microenvironment. On the contrary, FGL2 could increase the levels of tumoricidal cells such as CD8^+^ T cytotoxic cells, natural killer (NK) cells in the tumor microenvironment. So, FGL2 affect KRAS by influencing immune status in tumor environment of lung cancer. TP53 is regarded as tumor suppressor gene that it could prevent genome mutation. TP53 could activate DNA repair process and arrest cell proliferation by holding cell cycle  (Uehara & Tanaka, 2018). Mutation of TP53 leads to tumor escape from senescence and apoptosis. Activation of TP53 could increase the levels of tumor-infiltrating leukocytes such as CD8^+^ T cells in tumor microenvironment (Guo et al., 2017). FGL2 also increases the levels of tumor-infiltrating leukocytes such as CD8^+^ T cells in tumor microenvironment. So, FGL2 possesses synergic effects with TP53 to enhance antitumor immunity in tumor microenvironment. STK11, known as liver kinase B1 (LKB1), regulates cell polarity and regarded as tumor suppressor. Loss of STK11 would lead to cell polarity disorganization and induce tumor growth. Koyama et al. found that loss of STK11/LKB1 induced neutrophil recruitment and inflammatory mediator production to suppress the T cells in tumor environment of lung cancer (Koyama et al., 2016). In addition, STK11/LKB1 inactivated mutations were related with reduced expression of PD-1 ligand PD-L1 in tumor cells. So, we infer that FGL2 exert synergic effects with STK11 to enhance the cytotoxic T lymphocytes activities in tumor environment of lung adenocarcinoma. NF1 is regarded as a tumor suppressor in lung cancer negatively regulates RAS signaling pathway. NF1 mutations present in similar patients populations with KRAS mutation (Redig et al., 2016). We infer that FGL2 affect KRAS by influencing immune status in tumor environment of lung adenocarcinoma. So, FGL2 might affect NF1 function by influencing immune status in tumor environment of lung adenocarcinoma. KEAP1 has been shown to interact with nuclear factor erythroid 2-related factor 2 (NRF2). KEAP1-NRF2 pathway play important role in oxidative response by inducing anti-inflammatory and antioxidant effects. KEAP1 mutation is correlated with poor prognosis of lung cancer (Frank et al., 2018). Aberrant KEAP1-NRF2 pathway activity alters the immune microenvironment of lung adenocarcinoma. KEAP1 mutation is associated reduced leukocyte infiltration of tumor microenvironment in lung adenocarcinoma (Thorsson et al., 2018). We infer that FGL2 affects the KEAP1 effect by influencing immune status in tumor environment of lung adenocarcinoma. So, we infer some indirect correlations might explain the mechanism of FGL2 affecting other genes. FGL2 might affect other genes functions by influencing immune status in tumor environment of lung adenocarcinoma.

## Conclusion

In this study, we explored the expression profile and potential effects of *FGL2* in lung adenocarcinoma. We found that *FGL2* expression was significantly lower in lung adenocarcinoma tissue than in adjacent normal tissue. High *FGL2* mRNA expression was correlated with better prognostic outcomes of lung adenocarcinoma patients, including overall survival and progression-free survival. These results indicate that FGL2 might function as a negative regulator of lung adenocarcinoma. Then, we investigated the potential mechanism of FGL2 in lung adenocarcinoma. *FGL2* was positively correlated with the infiltration of immune cells, including CD8^+^ T cells, CD4^+^ T cells, macrophages, B cells and dendritic cells, in lung adenocarcinoma. These results imply that FGL2 exerts its antitumor effects by enhancing immune cell infiltration in lung adenocarcinoma. GO and KEGG functional enrichment analyses and GSEA also showed that *FGL2* expression was positively correlated with enhanced tumor killing. Thus, we propose that FGL2, which is positively associated with enhanced antitumor activities mediated by T cells, is a beneficial marker for lung adenocarcinoma treatment outcomes. In this study, we used bioinformatic analysis to discover the potential roles of FGL2 in lung adenocarcinoma. In the future studies, *in vivo* and *in vitro* experiments will carry out to demonstrate the role of FGL2 in modulating the T cell-mediated immune response in lung adenocarcinoma.

##  Supplemental Information

10.7717/peerj.8654/supp-1 Table S1 Basic information of included patients

10.7717/peerj.8654/supp-2 Supplemental Information 2 Code 1

10.7717/peerj.8654/supp-3 Supplemental Information 3 Code 2

## Additional Information and Declarations

### Data Availability

The following information was supplied regarding data availability: The code is available in the Supplemental Files.
