# Supplementary material for: FGL2 is positively correlated with enhanced antitumor responses mediated by T cells in lung adenocarcinoma
Source: PeerJ. 2020 Mar 13;8:e8654. doi: 10.7717/peerj.8654 (PMC7075367; doi:10.7717/peerj.8654)
Supplement: Table S1 [file peerj-08-8654-s001.docx]

**Supplementary Table. Basic information of included patients.**

| Database | Age (years) | Gender  (Male: Female) | Stage 1  (Male: Female) | Stage 2  (Male: Female) | Stage 3  (Male: Female) | Stage 4  (Male: Female) |
| --- | --- | --- | --- | --- | --- | --- |
| TCGA | 65.2±10.1 (33-88) | 244: 282 | 124: 164 | 67: 54 | 39: 46 | 12: 12 |
| PNAS | 63.2±10.2 (33-88) | 53: 72 | 32: 44 | 9: 15 | 4: 6 | 2: 1 |
| GEPIA | 65.2±10.1 (33-88) | 244: 282 | 124: 164 | 67: 54 | 39: 46 | 12: 12 |
| GSE13213 | 60.7±10.2 (39-86) | 60: 57 | 41: 38 | 9: 4 | 10: 15 | 0: 0 |
| GSE32863 | 67.8±9.6 (32-84) | 12: 46 | 8: 26 | 1: 10 | 3: 9 | 0: 1 |
| KM plotter, OS | None | 328: 287 | 159: 187 | 63: 55 | 12: 9 | 2: 2 |
| KM plotter, FP | None | 222: 221 | 127: 147 | 54: 44 | 7: 1 | 0: 0 |
